# Unnecessary magnetic resonance imaging of the knee: How much is it really costing the NHS?

**DOI:** 10.1016/j.amsu.2021.102736

**Published:** 2021-08-28

**Authors:** Muhammad Murtaza Khan, Bethan Pincher, Ricardo Pacheco

**Affiliations:** Northern Lincolnshire and Goole Foundation Trust, Scunthorpe General Hospital, DN15 7BH, UK

**Keywords:** National health services, Magnetic resonance imaging, Plain radiograph

## Abstract

**Aims and objectives:**

The aim of this study was to evaluate the indications for patients undergoing magnetic resonance imaging (MRI) of the knee prior to referral to an orthopaedic specialist and to ascertain whether these scans altered initial management.

**Materials and method:**

A retrospective review of all referrals received by a single specialist knee surgeon over a 1-year period was performed. Patient demographics, relevant history, examination findings and past surgical procedures were documented. Patients having undergone Magnetic resonance imaging (MRI) prior to referral were identified and indications for the scans recorded. These were reviewed against The National health services (NHS) guidelines for Primary Care Physicians to identify if the imaging performed was appropriate in each case.

**Results:**

A total of two sixty-one (261) patients were referred between 1^st^ July 2018 and 30^th^ June 2019. Eight seven out of two hundred and sixty-one patients (87/261) patients underwent knee MRI prior to referral. The average patient age was 53 years with male predominance (52 verses 35 females). Twenty-one out of eight seven patients under review (24%) underwent appropriate imaging prior to referral as per guidelines. However, only thirteen percent of patients underwent plain radiograph of knee before their scan. In cases where magnetic resonance imaging was not indicated, patients waited an average of twelve weeks between their scan and for a referral to be sent to a knee surgeon.

**Conclusion:**

Seventy six percent of patients referred to orthopaedics had inappropriate Magnetic resonance imaging arranged by their primary care physician. For a single consultant's referrals over 1 year these unnecessary MRI (magnetic resonance imaging) of knee cost National Health Services (NHS) £13,200. Closer adherence to the guidelines by primary care physicians will result in a financial saving, better patient experience and a more effective use of resources.

## Highlights

• Plain radiograph should be the first line of investigation for diagnosis of osteoarthritis.
• A good history covering nature, site of pain, locking and history of trauma should be considered.
• In patients with clear history of trauma and that are young then MRI should be considered or urgent referral to orthopedic specialty.
• Incidence of degenerative tear increases with age. The first line of management for degenerative tear is conservative treatment in terms of physiotherapy and pain relief.
•Plain radiograph particularly the weight bearing views can give more information in terms of joint space narrowing compared to MRI which is a static investigation.

## 1 Introduction

Knee joint is most commonly affected by osteoarthritis [1]. Non traumatic painful knee is a common complaint in outpatient knee clinic. The initial work up involves history, examination, and relevant investigation [2]. Plain radiographs are still considered as the first line investigation for diagnosis of osteoarthritis. Along with appropriate clinical examination, plain radiograph provides a sensitivity and specificity of 91% and 86% respectively for diagnosis of osteoarthritis [3].

Magnetic resonance imaging of knee provides excellent soft tissue contrast and allow accurate morphological assessment and it is the most comprehensive non-invasive investigation for knee joint [4]. Current systemic review and meta-analysis has revealed careful clinical correlation when interpreting MRI findings as it detects osteoarthritis at a relatively high rate in asymptomatic patients over age of 40 years by 19–43% [5]. The significance of magnetic resonance imaging findings in management of patients with reported early changes has been under review for clinical significance as toconsider them as part of normal aging process or label them as initial stage of osteoarthritis [6].

Along with detection of early osteoarthritis MRI is also an important diagnostic tool for detection of meniscal tear with sensitivity and specificity of 85–95%. Horizontal tears are considered as part of degenerative changes in the knee [4]. There is a growing consensus that these types of tear should be managed by non operative / conservative treatment as first modality of choice [7].

Longitudinal and vertical tear are associated with traumatic knee injury and are seen in young subjects. There is a strong association of anterior cruciate ligament injuries for these tears and are treated surgically [2].

The current technique of static imaging of MRI has been questioned by researchers as it is not able to provide the best physiological assessment of stresses on the knee joint in weight bearing position [1]. Moreover, patient factors like obesity and over-weight patients can also alter the interpretation of results due to increased physiological stress [8]. The overutilization of MRI has been reported in the literature by primary care physicians leading to poor utilization of funds and high rate of inappropriate imaging leading to no change in overall patient management [2].

Thus, MRI of knee should be requested in terms of clinical co-relation. Considering the above literature, we evaluated the indications for MRI of the knee joint requested by primary care physicians prior to referral to orthopedic consultant considering National Health Services Guidelines for General Physicians in United Kingdom [9].

## 2 Materials and Method

We retrospectively reviewed all referrals received by a single specialist knee surgeon over a 1-year period. Patient demographics, relevant history, examination findings and past surgical procedures were documented. Patients having undergone MRI prior to referral were identified and indications for the scans recorded. These were reviewed against The NHS guidelines for Primary Care Physicians to identify if the imaging performed was appropriate in each case. (Table 1).

**Table 1:** MRI contraindications as per NHS guidelines.

Age <15 years or over 45 years	Yes No
Locked knee 15° extension unable to flex to 90°	Yes No
Pseudo locking ---momentary period of stiffness following immobility	Yes No
Any osteoarthritis of knee	Yes No
Anterior knee pain due to OA, chondromalacia patella, tendon problem	Yes No
High BMI patient with radiological/clinical evidence of OA	Yes No
Previous meniscal surgery	Yes No
Active knee inflammatory arthritis	Yes No

The referred patients were broadly classified into two groups subjects with traumatic injury to the knee and non-traumatic painful knee (Table 2).

**Table 2:** Patient diagnosis as documented in referral.

Patient Diagnosis	
Knee pain	40
Knee pain and instability	11
Locked knee	5
Locked painful knee	1
Osteoarthritis	6
Painful meniscal injury	8
Meniscal injury	5
Traumatic Anterior Cruciate Ligament Injury	4
Patellar Dislocation	2
Others	5

As per NHS guidelines [9] a list of specific contraindications was developed where MRI was not indicated.

The project was registered as a retrospective review and 261 patients were evaluated that were referred to one knee consultant in Scunthorpe General Hospital. The study period was from 1^st^ July 2018 to 30^th^ June 2019. Delay between the MRI request and referral to the knee surgeon was noted along with time required for reporting the study. Moreover, the first choice of investigation I.e. plain radiograph or MRI of the knee was also taken into account. To evaluate the impact of investigation on patient care we also traced that whether MRI has resulted in change in plan of management of patient or not.

## 3 Results

**Fig. 1:**
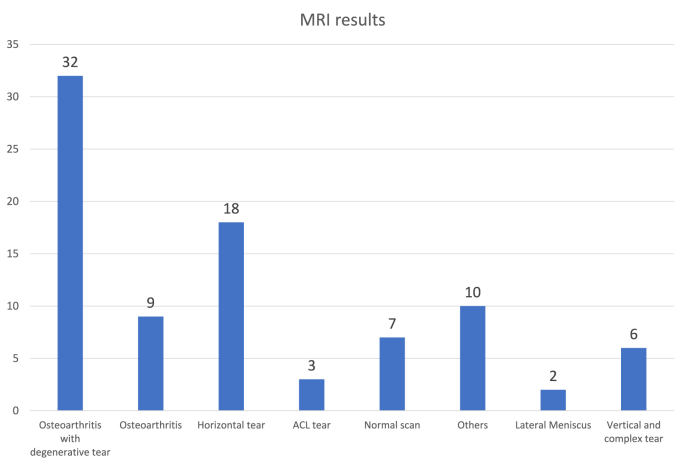
Results of MRI scan.

A total of 261 patients were referred during the study period. Eighty-seven out of two sixty one patients underwent MRI of the knee joint prior to referral. A brief description of MRI report is shown. (Fig. 1). The mean patient age was 53 years with predominance of male patients (52 verses 35 females). Twenty one out of eighty-seven (21/87) patients (24%) underwent the appropriate imaging prior to referral with only thirteen percent of patients underwent plain x-ray before their MRI. In cases where MRI was not indicated, as per guidelines patients ended up waiting for an average of 12 weeks between their scan and referral being sent to the specialist knee surgeon

Only a small proportion of patients in the study I.e. nine out of eight seven (0.87%) had traumatic injury to the knee. MRI led to change in management of patients with traumatic injury to knee and were offered arthroscopic repair of soft tissues injuries to knee. Seventy-eight out of eight seven patients (89%) had no significant history of trauma and presented with painful knee. The most common diagnosis that led to generation of referral to the specialist was anterior knee pain I.e. forty out of eight seven (45%).

MRI in non-traumatic group did not lead to change in management of patients and treatment option in terms of physiotherapy, intraarticular injections or knee arthroplasty were considered.

The brief summary of contraindications to MRI as per NHS guidelines can be seen in Table 1. The conclusions drawn following MRI scan report are shown in Fig. 1 Table 3 demonstrates the "number of contraindications" in our cohert of patients compared to standards as recommended by NHS guidlelines [9].

**Table 3:** No. of contraindication as per NHS guidelines.

MRI Contraindications (as per NHS guidelines document)	
5 contraindications	03
4 contraindications	09
3 contraindications	29
2 contraindications	14
1 contraindication	11

**Table 4:** Degenerative tear prevalence.

Prevalence of Meniscal tear with increasing age [2]	
50–59 years	25%
60–69 years	35%
70–79 years	45%
Osteoarthritis diagnosed on X ray	75–95%

In case of five out of eight seven patients referral was sent without waiting for the MRI to get reported by the radiologist. The quality of referral was also evaluated in terms of documentation of appropriate clinical findings. Thirty eight out of eighty-seven patients (43%) had appropriate clinical findings documented on the referral sent to the knee surgeon. We searched for two documents for this assessment i.e., both the original referral as well as the radiology request form.

## 4 Discussion

As per our study as well as evidence in the literature osteoarthritis usually affect individuals who are middle aged with females sometimes being affected more compared to men [2].

The degenerative changes in the knee are reported to arise in subjects over the age of 35 years particularly when there is no significant history of trauma. The age-related meniscal tear affects 50% of male patients with age between 70 and 90 years and 16% of female between 50 and 59 years of age [8].

MRI is not recommended for the diagnosis of osteoarthritis as it will not change the treatment planning. There is more chance of incidental finding of degenerative meniscal tear [7]. However, weight bearing radiographs of knee can give more information regarding reduced joint space.

In a study reported in literature 13% of patient had an MRI scan despite established diagnosis of osteoarthritis with the help of plain radiograph of knee [10].The use of MRI as a principal modality of choice for osteoarthritis is not supported in the literature and plain radiographs are still considered as first line of investigation especially in middle aged and elderly individuals.

Table 4 demonstrates increased prevelance of degenerative meniscal with increase in age. This resonates with MRI findings in our study which depicted evidence of degenerative tear with osteoarthritis in 32 subjects, followed by horizontal tear of medial meniscus in 18 patients. Horizontal tear of medial meniscus run parallel to the tibial surface and are usually degenerative in nature as defined in the reference study [4].

As per recent guidelines the management of degenerative meniscal tear did not result in superior functional outcome when treated with knee arthroscopy compared to conservative management [7].

Nine out of eight seven patients had history of recent trauma and presented with significant symptoms of knee pain and instability. MRI in this study group not only helped in diagnosis but also aided in pre-operative planning. This is again as reported in the literature which signifies the fact that traumatic soft tissue injuries should be treated with definitive management and are significant cause of patient symptoms. This group of subjects are usually young and are high demand individuals [10].

In one study the change in management was reported to be 26% following MRI scan for traumatic knee injury [2]. However, the use of MRI in non-traumatic knee pain has traditionally been reported to be less yielding when considering the change in management as an end point of investigation. Forty five percent (45%) of MRI requested by non-orthopedic physicians were either normal or just showing osteoarthritis compared to 27.6% when orthopedic physician were responsible for request. Similarly, the usefulness of MRI was highest in traumatic knee I.e., 84% verses degenerative knees 18% [3].

Considering the traditional low yield in terms of patient management various studies in literature have tried to raise awareness regarding MRI request and tried to establish an algorithim for requesting the scan. Another important aspect of this problem is to address the perception of patient about the investigation. This goes hand in hand with education of physicians requesting the scan [2].

Literature also supports the idea of interpretation of findings in relation to clinical picture. MRI can detect cartilage damage, osteophytes in 87% of patients without any risk factor for developing of advanced osteoarthritis in one study [6].

Another limiting factor regarding the results of MRI scan is the overweight patients with high BMI, the underlying reason for this variation is the fact that there is increased strain on the tibiofemoral cartilage leading to decrease in thickness leading to altering the results of scan [8].

Obese and overweight patients are also considered as a poor candidate for knee arthroplasty. Thus, the use of MRI in over-weight patients again will not lead to change in management in most cases. As finding of degenerative tear will not make them candidate for arthroscopy. Moreover, as per evidence these patients do poorly after arthroplasty as well thus making them a poor surgical candidate in general.

The limitations of our study are that it is a single centre, retrospective study and we evaluated the referral sent to only one knee consultant working in the Trust. However, considering the magnitude of problem we believe that there is an urgent need to raise awareness in primary care physicians regarding the guidelines and criteria for requesting MRI scan.

## 5 Conclusion

1. Seventy six percent of patients referred to orthopaedics had inappropriate Magnetic resonance imaging arranged by their primary care physician. For a single consultant's referrals over 1 year these unnecessary MRI (magnetic resonance imaging) of knee cost National Health Services (NHS) £13,200. Closer adherence to the guidelines by primary care physicians would result in a financial saving, faster referral times and a more effective use of resources.
2. Implementation of guidelines and better understanding of use of MRI as a modality of choice can help in preventing unnecessary delays in treatments especially for soft tissues injuries of the knee where delay can lead to poor prognosis and prolonged period of rehabilitation.
3. The study also stresses the need of time where primary care and orthopedic surgeons should work in a more interactive and dynamic way to streamline patient care thus contributing to better understanding of routine/urgent referral and investigation protocol system.

## Ethical approval

The project was registered as an audit after discussion with the local research team. The approval was taken from all the consultants in the local hospital and findings were discussed with the local Clinical commissioning group as well as in the department audit meeting.

## Sources of funding

There is no source of funding used for this project.

## Author contribution

Muhammad Murtaza khan: Manuscript writing, data collection, evaluation, review of literature.

Bethan Pincher: Peer review.

Ricardo Pacheco: main idea, lead consultant.

## Registration of research studies

1. Name of the registry:
2. Unique Identifying number or registration ID:
3. Hyperlink to your specific registration (must be publicly accessible and will be checked):

## Consent

None.

## Guarantor

Muhammad Murtaza Khan.

I take full responsibility of this work. I have handled the data and to the best of my knowledge the information provided is correct.

## Declaration of competing interest

There is no conflict of interest associated with this study.
