# Supplementary material for: Unnecessary magnetic resonance imaging of the knee: How much is it really costing the NHS?
Source: Ann Med Surg (Lond). 2021 Aug 28;70:102736. doi: 10.1016/j.amsu.2021.102736 (PMC8463827; doi:10.1016/j.amsu.2021.102736)
Supplement: Multimedia component 1 [file mmc1.pptx]

## Slide 1
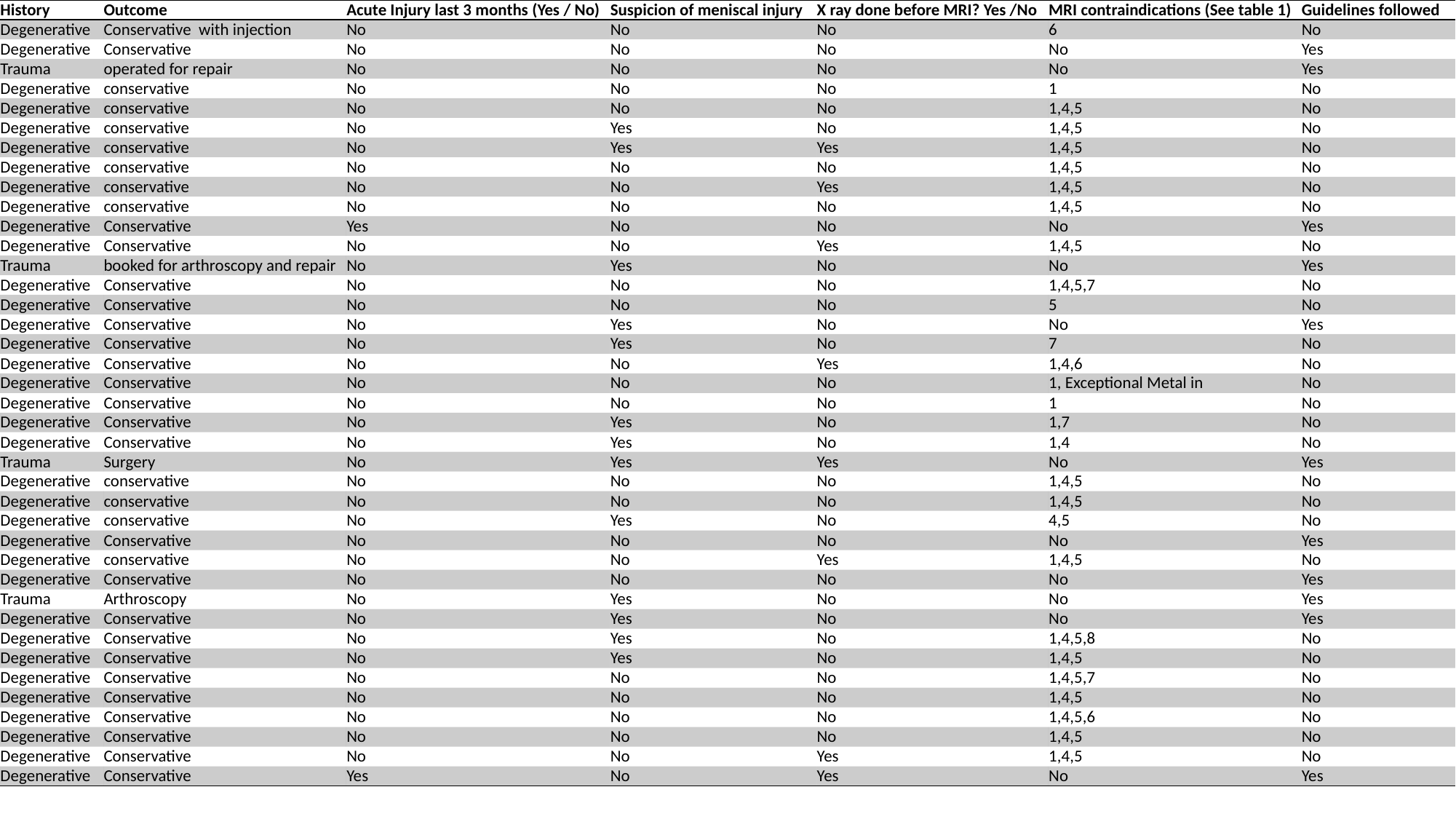

| History | Outcome | Acute Injury last 3 months (Yes / No) | Suspicion of meniscal injury | X ray done before MRI? Yes /No | MRI contraindications (See table 1) | Guidelines followed |
| --- | --- | --- | --- | --- | --- | --- |
| Degenerative | Conservative with injection | No | No | No | 6 | No |
| Degenerative | Conservative | No | No | No | No | Yes |
| Trauma | operated for repair | No | No | No | No | Yes |
| Degenerative | conservative | No | No | No | 1 | No |
| Degenerative | conservative | No | No | No | 1,4,5 | No |
| Degenerative | conservative | No | Yes | No | 1,4,5 | No |
| Degenerative | conservative | No | Yes | Yes | 1,4,5 | No |
| Degenerative | conservative | No | No | No | 1,4,5 | No |
| Degenerative | conservative | No | No | Yes | 1,4,5 | No |
| Degenerative | conservative | No | No | No | 1,4,5 | No |
| Degenerative | Conservative | Yes | No | No | No | Yes |
| Degenerative | Conservative | No | No | Yes | 1,4,5 | No |
| Trauma | booked for arthroscopy and repair | No | Yes | No | No | Yes |
| Degenerative | Conservative | No | No | No | 1,4,5,7 | No |
| Degenerative | Conservative | No | No | No | 5 | No |
| Degenerative | Conservative | No | Yes | No | No | Yes |
| Degenerative | Conservative | No | Yes | No | 7 | No |
| Degenerative | Conservative | No | No | Yes | 1,4,6 | No |
| Degenerative | Conservative | No | No | No | 1, Exceptional Metal in | No |
| Degenerative | Conservative | No | No | No | 1 | No |
| Degenerative | Conservative | No | Yes | No | 1,7 | No |
| Degenerative | Conservative | No | Yes | No | 1,4 | No |
| Trauma | Surgery | No | Yes | Yes | No | Yes |
| Degenerative | conservative | No | No | No | 1,4,5 | No |
| Degenerative | conservative | No | No | No | 1,4,5 | No |
| Degenerative | conservative | No | Yes | No | 4,5 | No |
| Degenerative | Conservative | No | No | No | No | Yes |
| Degenerative | conservative | No | No | Yes | 1,4,5 | No |
| Degenerative | Conservative | No | No | No | No | Yes |
| Trauma | Arthroscopy | No | Yes | No | No | Yes |
| Degenerative | Conservative | No | Yes | No | No | Yes |
| Degenerative | Conservative | No | Yes | No | 1,4,5,8 | No |
| Degenerative | Conservative | No | Yes | No | 1,4,5 | No |
| Degenerative | Conservative | No | No | No | 1,4,5,7 | No |
| Degenerative | Conservative | No | No | No | 1,4,5 | No |
| Degenerative | Conservative | No | No | No | 1,4,5,6 | No |
| Degenerative | Conservative | No | No | No | 1,4,5 | No |
| Degenerative | Conservative | No | No | Yes | 1,4,5 | No |
| Degenerative | Conservative | Yes | No | Yes | No | Yes |
