# Supplementary material for: Unnecessary magnetic resonance imaging of the knee: How much is it really costing the NHS?
Source: Ann Med Surg (Lond). 2021 Aug 28;70:102736. doi: 10.1016/j.amsu.2021.102736 (PMC8463827; doi:10.1016/j.amsu.2021.102736)
Supplement: Multimedia component 2 [file mmc2.pptx]

## Slide 1
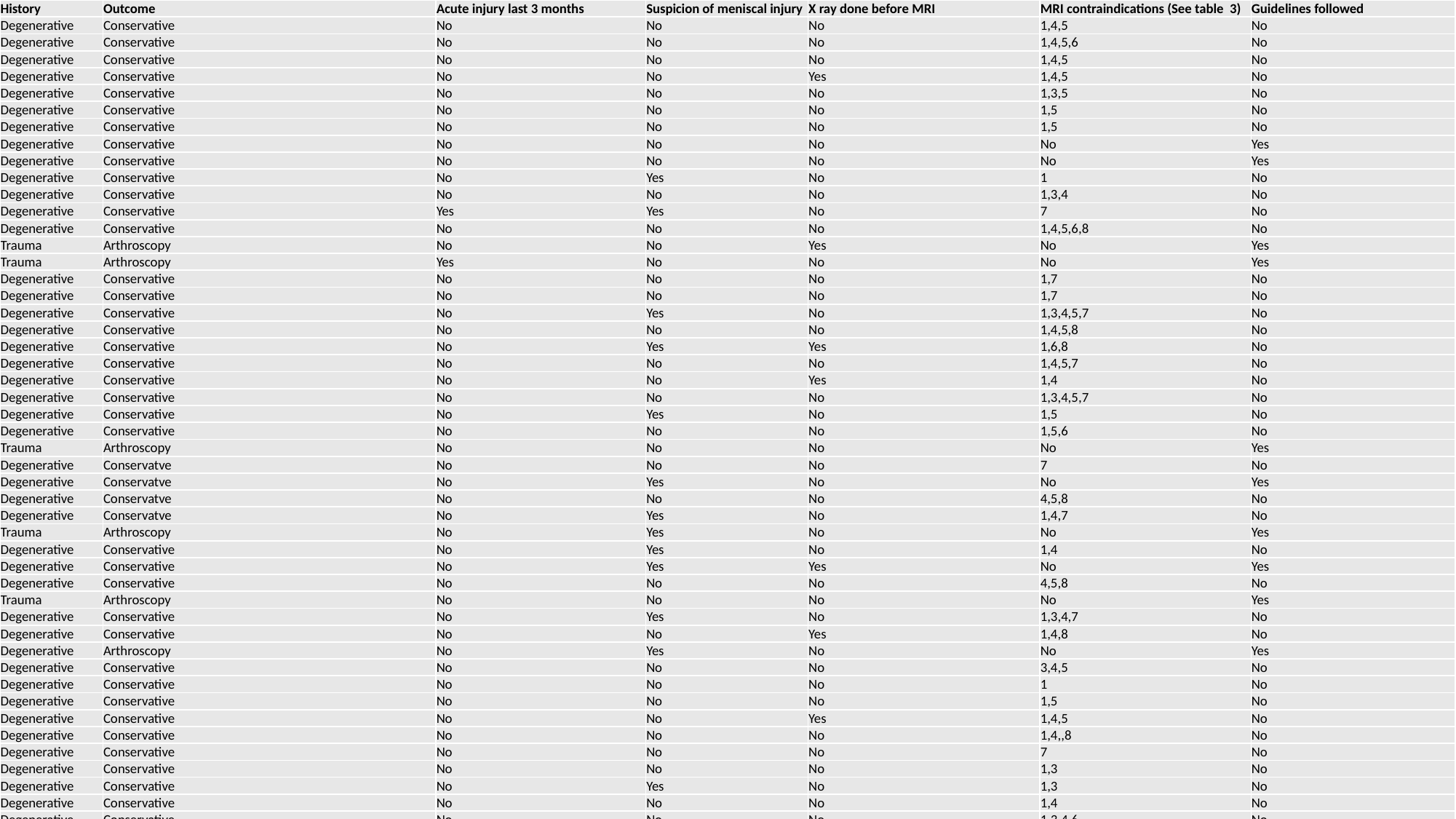

| History | Outcome | Acute injury last 3 months | Suspicion of meniscal injury | X ray done before MRI | MRI contraindications (See table 3) | Guidelines followed |
| --- | --- | --- | --- | --- | --- | --- |
| Degenerative | Conservative | No | No | No | 1,4,5 | No |
| Degenerative | Conservative | No | No | No | 1,4,5,6 | No |
| Degenerative | Conservative | No | No | No | 1,4,5 | No |
| Degenerative | Conservative | No | No | Yes | 1,4,5 | No |
| Degenerative | Conservative | No | No | No | 1,3,5 | No |
| Degenerative | Conservative | No | No | No | 1,5 | No |
| Degenerative | Conservative | No | No | No | 1,5 | No |
| Degenerative | Conservative | No | No | No | No | Yes |
| Degenerative | Conservative | No | No | No | No | Yes |
| Degenerative | Conservative | No | Yes | No | 1 | No |
| Degenerative | Conservative | No | No | No | 1,3,4 | No |
| Degenerative | Conservative | Yes | Yes | No | 7 | No |
| Degenerative | Conservative | No | No | No | 1,4,5,6,8 | No |
| Trauma | Arthroscopy | No | No | Yes | No | Yes |
| Trauma | Arthroscopy | Yes | No | No | No | Yes |
| Degenerative | Conservative | No | No | No | 1,7 | No |
| Degenerative | Conservative | No | No | No | 1,7 | No |
| Degenerative | Conservative | No | Yes | No | 1,3,4,5,7 | No |
| Degenerative | Conservative | No | No | No | 1,4,5,8 | No |
| Degenerative | Conservative | No | Yes | Yes | 1,6,8 | No |
| Degenerative | Conservative | No | No | No | 1,4,5,7 | No |
| Degenerative | Conservative | No | No | Yes | 1,4 | No |
| Degenerative | Conservative | No | No | No | 1,3,4,5,7 | No |
| Degenerative | Conservative | No | Yes | No | 1,5 | No |
| Degenerative | Conservative | No | No | No | 1,5,6 | No |
| Trauma | Arthroscopy | No | No | No | No | Yes |
| Degenerative | Conservatve | No | No | No | 7 | No |
| Degenerative | Conservatve | No | Yes | No | No | Yes |
| Degenerative | Conservatve | No | No | No | 4,5,8 | No |
| Degenerative | Conservatve | No | Yes | No | 1,4,7 | No |
| Trauma | Arthroscopy | No | Yes | No | No | Yes |
| Degenerative | Conservative | No | Yes | No | 1,4 | No |
| Degenerative | Conservative | No | Yes | Yes | No | Yes |
| Degenerative | Conservative | No | No | No | 4,5,8 | No |
| Trauma | Arthroscopy | No | No | No | No | Yes |
| Degenerative | Conservative | No | Yes | No | 1,3,4,7 | No |
| Degenerative | Conservative | No | No | Yes | 1,4,8 | No |
| Degenerative | Arthroscopy | No | Yes | No | No | Yes |
| Degenerative | Conservative | No | No | No | 3,4,5 | No |
| Degenerative | Conservative | No | No | No | 1 | No |
| Degenerative | Conservative | No | No | No | 1,5 | No |
| Degenerative | Conservative | No | No | Yes | 1,4,5 | No |
| Degenerative | Conservative | No | No | No | 1,4,,8 | No |
| Degenerative | Conservative | No | No | No | 7 | No |
| Degenerative | Conservative | No | No | No | 1,3 | No |
| Degenerative | Conservative | No | Yes | No | 1,3 | No |
| Degenerative | Conservative | No | No | No | 1,4 | No |
| Degenerative | Conservative | No | No | No | 1,3,4,6 | No |
